# Enzymes (α-Amylase, Xylanase, and Cellulase) in Steamed Buckwheat Buns: The Effects on Quality and Predicted Glycemic Response

**DOI:** 10.3390/foods14152735

**Published:** 2025-08-05

**Authors:** Wenjun Liu, Jian Ming, Margaret Brennan, Charles Brennan

**Affiliations:** 1 College of Food Science, Southwest University, Chongqing 400715, China; 18580612528@163.com; 2 Chongqing Academy of Chinese Materia Medica, Chongqing 400065, China; 3 Chongqing Wanbiao Testing Technology Ltd., Chongqing 400714, China; 4 School of Science, RMIT, Melbourne, VIC 3000, Australia; margaretinpalmy@yahoo.co.nz

**Keywords:** pseudocereal, dietary fiber, physicochemical properties, in vitro digestion, synergistic mechanism

## Abstract

This study investigated the individual and combined effects of α-amylase (6 and 10 ppm), xylanase (70 and 120 ppm), and cellulase (35 and 60 ppm) on the physicochemical and nutritional properties of Chinese steamed buns (CSBs) supplemented with 15% buckwheat flour. The addition of individual enzymes did not significantly affect the volume or texture of the buckwheat-enriched CSBs, although it increased the crumb moisture content and porosity. In contrast, enzyme combinations can improve specific volume and reduce hardness. The enzyme combination (α-amylase 6 ppm, xylanase 70 ppm, and cellulase 60 ppm) yielded the highest specific volume (2.50 mL/g) and the lowest hardness (271.46 g). Regarding chemical properties, individual enzymes had minimal impact, while the combined treatment (6, 70, 60 ppm) decreased starch and dietary fiber content. For nutritional properties, the glycemic response of the CSBs varied depending on the concentration of the enzyme combination used.

## 1. Introduction

Buckwheat, a pseudocereal of the Polygonaceae family in the genus Fagopyrum, is widely cultivated across major producing regions, including China, Russia, Canada, the United States, and Italy [1,2]. According to research, buckwheat grain is rich in valuable nutritional components, including dietary fiber (DF), proteins, vitamins, and mineral elements [3,4]. Bioactive compounds in buckwheat have demonstrated various physiological activities in vitro and in vivo (animal models), such as potential cholesterol-lowering, anti-inflammatory, anti-glycemic, and anti-hypertensive effects, as well as anticancer activity [5,6,7]. While these findings highlight the potential health-promoting properties of buckwheat, more clinical research is needed to substantiate these effects and establish their impact in human populations. As a good source of DF, buckwheat serves as a functional ingredient in diverse food products, including buns, noodles, cakes, and extruded snacks [8].

Chinese steamed buns (CSBs), also known as mantou, is a traditional fermented staple food, accounting for approximately 40% of the wheat consumption in China [9,10]. In our previous research, steamed buns incorporated with 15% buckwheat flour led to a reduction in predicted glycemic response [11]. Additionally, Liu et al. [12,13] indicated that the addition of buckwheat into normal bun dough has detrimental effects on dough rheology and the quality of the CSBs, such as reducing the volume of the buns, darkening the crumb appearance, and increasing the firmness of the buns.

In order to enhance the quality of buckwheat-enriched CSBs, enzymes such as amylases, cellulases, and xylanases can be incorporated as processing aids during bread making. Fungal α-amylase hydrolyzes α-1,4-glycosidic bonds in starch, producing maltodextrins and oligosaccharides. [14]. Its widespread application in the food industry leverages this activity to enhance processing efficiency and product quality [15]. Xylanase randomly attacks the arabinoxylan (AX) backbone and breaks glycosidic linkages in AX, resulting in altered functional and physicochemical properties of AX [16]. Cellulase, classified under glycoside hydrolases, hydrolyzes β-1,4-glucosidic linkages in cellulose and related β-D-glucans via synergistic action of endoglucanases, exoglucanases, and β-glucosidases [17]. These three classes of commercial enzyme preparations dominate the baking industry, collectively improving the rheological behavior of dough and hence the quality of the final products [18,19]. Nevertheless, the synergistic impact of enzyme combinations on dough rheology, bun quality parameters, and predicted glycemic response is inadequately documented.

The aim of this study was to investigate the effect of α-amylase, xylanase, and cellulase, added at varying concentrations, on the quality and in vitro glycemic response of Chinese steamed buns (CSBs) containing 15% buckwheat flour.

## 2. Materials and Methods

### 2.1. Ingredients

The ingredients for the Chinese steamed buns consisted of wheat flour (Champion Flour Milling Ltd., Christchurch, New Zealand), buckwheat flour (Ceres Organics Ltd., Auckland, New Zealand), yeast powder, and salt (Pams Products Ltd., Auckland, New Zealand).

The following Novozymes (Sydney, Australia) enzyme preparations were used: Fungamyl 2500 SG (fungal α-amylase, EC 3.2.1.1), Pentopan Mono BG (fungal xylanase, EC 3.2.1.8), and Cellulast BG (endo-glucanase, EC 3.2.1.4). Enzyme activities are listed in Supplementary Table S1.

### 2.2. Production of Chinese Steamed Buns

The base recipe contained wheat flour (200 g), yeast powder (4 g), salt (1 g), and water (adjusted to 500 FU maximum consistency). For experimental variants, wheat flour was partially replaced with 15% *w*/*w* wheat bran (WB) based on dry weight (15 g/100 g flour). Liquid enzyme preparations were incorporated during dough mixing. Post-fermentation, the dough was rolled out and proofed at 30 °C for 25 min. The proofed dough pieces were then steamed for 20 min. Finally, the steamed buns were cooled to room temperature prior to analysis.

### 2.3. Physical Properties of CSBs

The moisture content of CSBs followed AACC Method 44-16.01 via atmospheric oven drying (105 ± 2 °C, ≥12 h). Samples were dried at 105 ± 2 °C until constant mass, and results were calculated as grams of water per 100 g of sample [20]. Triplicate measurements were conducted to ensure reproducibility.

The specific volumes of the steamed buns were determined in triplicate via rapeseed displacement according to AACC International Approved Method 10-05.01 [20]. Triplicate measurements were conducted to ensure reproducibility.

The textural properties of the steamed buns were quantified via Texture Profile Analysis (TPA) using a TA-XT2 Texture Analyser (Stable Micro Systems, Surrey, UK) fitted with a 25 mm cylindrical probe. Samples were sectioned into 25 mm thick slices and subjected to two-cycle compression to simulate oral mastication behavior. Test parameters included pre-test speed, 1.0 mm/s; test speed, 1.7 mm/s; post-test speed, 10.0 mm/s; target strain, 40%; and trigger force, 5 g [13,21]. Triplicate measurements ensured statistical reliability.

The crumb porosity of the steamed buns was analyzed using image analysis according to Shibata’s method [22]. Bun slices were imaged from 5 cm using a Sony Digital 8 camera (Sony, Tokyo, Japan), capturing 24-bit BMP images (640 × 480 pixels) for storage. Subsequent analysis used ImageJ 1.51j8 (Bethesda, MD, USA) to convert images to 8-bit grayscale (0–255). Triplicate measurements were conducted to ensure reproducibility.

### 2.4. Starch and Fiber Content Analysis

Total starch content in the steamed buns was quantified using the Megazyme Total Starch Assay Kit (K-TSTA, Megazyme International, Wicklow, Ireland) according to the AACC Method, with modifications for small-scale DMSO solubilization as described by Brennan et al. [23].

Total, soluble (SDF), and insoluble dietary fiber (IDF) in the steamed buns supplemented with 15% buckwheat were quantified using the Megazyme Total Dietary Fibre Assay Kit (Megazyme International, Wicklow, Ireland) according to the AACC standard method [20].

### 2.5. Glycemic Response Analysis

In vitro glycemic response was measured following a modified method by Brennan et al. [24]. Triplicate-milled samples were suspended in 30 mL water and heated by stirring. Concurrently, 0.8 mL of 1 M HCl was added to sample tubes and vortexed. Following incubation at 37 °C, 1 mL of pepsin solution (10% in 0.05 M HCl) was added. Gastric digestion proceeded for 30 min at 37 °C with constant stirring. Subsequently, 2 mL of 1 M NaHCO_3_ and 5 mL of 0.1 M sodium maleate buffer (pH 6.0) were added. A 1 mL aliquot (0 min sample) was immediately quenched in 4 mL ethanol. Starch digestion was initiated by rapid sequential addition of 0.1 mL amyloglucosidase and 5 mL of a pancreatin solution (2.5% in 0.1 M sodium maleate buffer, pH 6.0). Incubation continued at 37 °C for 120 min with constant agitation. Additional 1 mL aliquots were quenched in triplicate at 20, 60, and 120 min using 4 mL of ethanol. Reducing sugars (expressed as glucose equivalents, GEs) were quantified via DNS assay following secondary enzymatic digestion with invertase and amyloglucosidase. Glucose standards were used for calibration, with all measurements performed in triplicate. The in vitro glycemic response was calculated as the Hydrolysis Index (HI), defined as the ratio of the Area Under the Curve (AUC) of glucose release (0–120 min) for the sample to that of a reference carbohydrate, multiplied by 100.

### 2.6. Design of Experiment

Two experimental designs were conducted: the first assessed individual enzyme effects on the physicochemical characteristics of the CSBs containing 15% buckwheat flour, while the second examined enzyme mixture effects. For the single enzyme, ANOVA analyzed physicochemical characteristics of regular CSBs and buckwheat-enriched CSBs (15% buckwheat control). Enzymes (Cellulast BG, Fungamyl 2500 SG, Pentopan Mono BG) (Novozymes, North Rocks, NSW, Australia) were dosed at 35 ppm, 10 ppm, and 70 ppm, respectively, following manufacturer recommendations (Novozymes) (Supplementary Table S1). For enzyme mixtures, a 2^3^ full-factorial design evaluated the main effects and second-order interactions of α-amylase, xylanase, and cellulase in CSBs incorporated with 15% buckwheat flour [25,26]. Three factors tested at two coded levels (−1, +1) generated eight experimental combinations, with level specifications detailed in Supplementary Table S2.

Based on estimated coefficients (βᵢ, βᵢⱼ, βᵢⱼₖ), the theoretical response variable (W) was calculated using the polynomial model, as follows:

(1) $$w=\beta_{0}+\Sigma \beta_{i}x_{i}+\Sigma \beta_{ij}\chi_{i}x_{j}+\beta_{123}ABC$$

Factors: A (α-amylase), B (xylanase), C (cellulase); Interactions: AB (A × B), AC (A × C), BC (B × C), ABC (A × B × C); Coefficients: β_0_ (global mean), βᵢ (main effects), βᵢⱼ/βᵢⱼₖ (interaction effects).

The regression defines relationships between physicochemical parameters and the three enzyme activities.

### 2.7. Statistical Analysis

ANOVA and multiple regression analysis were performed on all data using Minitab 17 statistical software (version 17.2.1; Minitab Pty Ltd., Sydney, Australia), with significance set at *p* < 0.05.

## 3. Results and Discussion

### 3.1. Effect of a Single Enzyme on Physical and Chemical Properties of CSBs Incorporated with 15% Buckwheat Flour

Table 1 presents the impact of individual enzymes on buckwheat-enriched CSBs’ physicochemical properties. α-Amylase supplementation (10 ppm) showed no significant effect on loaf volume but increased moisture content, chewiness, and crumb porosity. Chemical parameters remained statistically unchanged compared to control buckwheat-enriched CSBs. Chen et al. [27] similarly documented marginally increased (*p* > 0.05) specific volume in maltogenic amylase-supplemented buns. According to Motaharet al. [18], gluten-free bread with acidic, thermostable α-amylase exhibited a softer crumb, greater porosity, elevated moisture content, and darker crust coloration. Moreover, Lagrain et al. [28] showed that amylases had little impact on the bun volume. On the contrary, our previous research found the addition of 10 ppm α-amylase led to an increase in specific volume and a decrease in the hardness of CSBs enriched with 15% oat bran [13]. These inconsistent observations may be attributed to the distinct ways in which different amylases affect starch granules [29,30]. The extent of amylose and amylopectin hydrolysis by specific amylases determines yeast activity by influencing the availability of low-molecular-weight dextrins and short-chain unbranched polymers [31,32]. Furthermore, amylase concentration and flour type are also key factors contributing to the observed differences.

Regarding xylanase and cellulase, the physical parameters of control CSBs and 15% buckwheat-enriched CSBs showed no significant differences. However, enzyme addition significantly increased both porosity and moisture in the 15% buckwheat-enriched CSBs. Similar results were observed by Mohammadi et al. [33], who reported that the treatment containing 0.3 g xylanase in 100 g wheat flour had no significant effects on the volume and texture of baguette buns, whereas it increased the moisture and porosity of crumb. In addition, Mohammadi et al. [33] found that the addition of xylanase and pentosanase enzymes did not significantly affect baking uniformity, physical shape, taste, or odor. Our findings further suggested significantly low and declining activities of cellulase and xylanase throughout buckwheat dough fermentation. This phenomenon is potentially attributable to the specific activity and concentration of the enzymes used [34,35].

Compared to the control, the chemical properties of buckwheat buns containing single enzymes were not significantly altered, potentially due to insufficient activity of these enzymes during fermentation in the 15% buckwheat flour dough.

### 3.2. Effect of Enzyme Combinations on the Physical Properties of CSBs

The impact of combined enzymes on the physical properties of buckwheat-enriched CSBs was assessed via a 2^3^ full factorial design. Table 2 and Table 3 display the resulting regression coefficients and R^2^ values, leading to the following final empirical models for specific volume, moisture, hardness, springiness, chewiness, cells, cell size, and cell area.

(2) W (Specific volume) = 2.26 − 0.02A − 0.05B − 0.07C + 0.02AB + 0.03AC + 0.01BC + 0.02ABC (R^2^ = 0.98)

(3) W (Moisture) = 45.65 + 0.33A + 0.47B + 0.56C − 1.96AB + 0.66AC + 0.26BC − 0.44ABC (R^2^ = 0.99)

(4) W (Hardness) = 331.68 + 18.20A + 11.33C − 2.14AB − 6.69AC − 11.74BC − 13.21ABC (R^2^ = 0.98)

(5) W (Springiness) = 0.96 − 0.01A − 0.02B − 0.01C + 0.02AB + 0.01ABC (R^2^ = 0.90)

(6) W (Chewiness) = 308.18 + 23.53A + 8.52B + 11.31C − 3.21AB − 12.11AC − 3.08BC − 18.22ABC (R^2^ = 0.99)

(7) W (Cell density) = 49.52 + 1.86A + 1.26B − 1.81C + 3.66AB − 1.66BC (R^2^ = 0.91)

(8) W (Cell size) = 0.58 − 0.11A − 0.06B − 0.03AB + 0.03AC + 0.07BC − 0.03ABC (R^2^ = 0.97)

(9) W (Cell area) = 22.88 − 0.68A − 0.56B − 0.33C + 0.88AB + 0.27AC + 0.33BC (R^2^ = 0.90)

Regarding specific volume, α-amylase, cellulase, and xylanase individually exerted a negative effect on CSB volume. In contrast, the interactions between α-amylase–xylanase and α-amylase–cellulase demonstrated positive synergistic effects. This synergy resulted in increased specific volume with higher levels of the α-amylase × cellulase, α-amylase × xylanase, and xylanase × cellulase interaction terms. Consequently, compared to individual enzymes, enzyme combinations can improve the specific volume from 2.19 mL/g to 2.50 mL/g. Hmad et al. [36] also observed synergistic improvements in bread dough rheology and properties using a cocktail of α-amylase, xylanase, and cellulase, aligning with our findings. Additionally, Xue et al. [37] reported that combining xylanase and arabinofuranosidase synergistically enhanced wheat bran hydrolysis and the yield of soluble arabinoxylan xylooligosaccharides, improving dough rheology, as well as the nutritional and quality characteristics of steamed bread. These observations may relate to the synergistic mechanism between xylanase and cellulase [36,38]. Furthermore, our study demonstrated the synergistic effects of enzyme combinations on both loaf height and moisture content.

The enzyme cocktail (α-amylase, xylanase, cellulase) enhanced the textural properties of 15% buckwheat-enriched CSBs compared to individual enzyme treatments (Table 3). However, this combination exhibited detrimental synergism, adversely increasing both hardness and chewiness. According to the research of Kostyuchenko et al. [39], the bread formulated with an α-amylase, endo-xylanase, and exoprotease combination exhibited increased specific volume, porosity, and aldehyde content, but decreased shape stability, relative to the control without enzymes. Huang et al. [40] demonstrated that the inclusion of amylase, protease, and lipase significantly increased bread’s specific volume while simultaneously decreasing crumb hardness, moisture migration rate, and staling rate. Furthermore, the enzyme cocktail enhanced textural properties in 15% quinoa-enriched buns through targeted disruption of the starch–protein matrix [41].

For the crumb structure, the synergistic action of α-amylase, xylanase, and cellulase enhanced crumb porosity in CSBs containing 15% buckwheat flour. As a result, the effect of enzyme mixtures decreased the cell density from 51.35 to 46.68 cells/cm^2^, whereas it increased the cell size and cell area from 0.39 mm and 20.12% to 0.70 mm and 26.98%. Table 3 indicates that the α-amylase–xylanase interaction enhanced both cell density and cell area but reduced cell size. Conversely, the α-amylase–cellulase and xylanase–cellulase interactions increased cell size and cell area. However, the xylanase–cellulase combination decreased cell density. Consistent with these findings, Ebling et al. [42] reported that the synergistic effect of laccase and transglutaminase provoked structural reorganization, yielding buns with less cell density but a bigger cell area than those obtained by the treatment with transglutaminase alone due to gluten matrix loosening via disulfide bond redistribution. Matsushita et al. [43] illustrated that combining bakery enzymes can improve the formation of gluten networks in the dough, resulting in a bigger crumb cell than that obtained via the use of a single enzyme. Moreover, Sadeghian Motahar et al. [44] demonstrated that the addition of enzyme mixtures can improve the porosity, specific volume, and sensory of buns enriched with quinoa protein.

### 3.3. Effect of Enzyme Combinations on the Chemical Properties of CSBs

The combined effects of α-amylase, xylanase, and cellulase on the chemical properties of buckwheat-enriched CSBs are shown in Table 4 and Table 5. The application of α-amylase, xylanase, and cellulase collectively diminished dietary fiber (DF) and total starch content in buckwheat-enriched CSBs. The enzyme blend proved more effective than any single enzyme, minimizing both DF and starch levels. Park et al. [45] similarly observed that enzyme mixtures led to a reduction in both soluble and insoluble fiber content in buns, owing to the enzymatic hydrolysis of these components. According to the research of Matsushita et al. [43], an optimal combination of bakery enzymes resulted in lower concentrations of damaged starch and insoluble dietary fiber compared to control buns. Jagelaviciute et al. [46] also reported that enzyme combinations decreased the SDF and IDF content of buns supplemented with apple pomace extracts. This observation is likely attributable to the synergistic mechanism of α-amylase, xylanase, and cellulase during fermentation, wherein enzymatic interactions selectively hydrolyze complex polysaccharides.

With respect to the glycemic response, the AUC values were varied when the combination of enzymes was added at different concentrations. Consistent with our previous findings, the glycemic response of CSBs containing 15% wheat bran was modulated by varying concentrations of enzyme combinations. Chauhan et al. [47] demonstrated the synergistic action of a cellulase and amylase blend, which reduced protein, fat, and starch content while concurrently elevating both insoluble and soluble fiber fractions. Arte et al. [48] demonstrated that hydrolytic enzymes elevated reducing sugars and water-extractable (WE) pentosan content in wheat bran. These findings may elucidate variations in glycemic impact through enzymatic degradation and differential sugar release. Current studies reveal that dietary fiber forms protein-complexed matrices encapsulating starch granules, thereby inhibiting enzymatic activity [49,50]. Nevertheless, enzyme combinations disrupt this fiber–protein network via synergistic hydrolysis. Research on the effect of enzyme combinations on the glycemic response are scarce.

In summary, the synergistic mechanism involves coordinated multi-substrate targeting; glucose oxidase catalyzes interchain disulfide bond formation within glutenin macropolymers, while concomitant protease activity modulates gliadin hydrolysis to optimize dough extensibility and elastic recovery. Critically, reaction products may function as substrates for subsequent enzymatic reactions or as effectors modifying the reaction microenvironment. Exemplifying this cascade, xylanase-mediated arabinoxylan hydrolysis enhances gluten matrix hydration, thereby facilitating secondary enzymatic processes; similarly, lipase-generated monoglycerides synergize with amylase through the competitive inhibition of starch retrogradation [51,52,53,54].

## 4. Conclusions

This study systematically evaluated individual and synergistic impacts of α-amylase, xylanase, and cellulase on the physicochemical traits and nutritional profile of Chinese steamed buns (CSBs) formulated with 15% buckwheat flour substitution. The addition of individual enzymes did not significantly alter the volume or texture of buckwheat-enriched CSBs, although it elevated crumb moisture content and porosity. In contrast, enzyme combinations synergistically enhanced specific volume and cell size while reducing hardness, attributable to enzymatic interactions. Notably, the enzyme combination (α-amylase 6 ppm, xylanase 70 ppm, cellulase 60 ppm) improved the quality of buckwheat-enriched CSBs and exhibited negligible effects on the glycemic response. Consequently, this enzyme formulation demonstrates considerable industrial potential for baking applications.

## Figures and Tables

**Table 1:** Effect of single enzymes on the physical and chemical properties of CSBs.

Bun Samples	Wheat Flour	15% Buckwheat (Control)	15% Buckwheat + 10 ppm Amylase	15% Buckwheat + 70 ppm Xylanase	15% Buckwheat + 35 ppm Cellulase
Specific volume (mL/g)	2.50 ± 0.03 A	2.19 ± 0.01 B	2.20 ± 0.01 B	2.20 ± 0.01 B	2.22 ± 0.01 B
Loaf height (mm)	62.14 ± 0.38 A	57.65 ± 0.30 B	57.31 ± 0.31 B	56.46 ± 0.68 B	56.91 ± 0.88 B
Moisture (%)	40.10 ± 0.01 D	41.15 ± 0.10 C	45.93 ± 0.21 A	42.68 ± 0.29 B	45.91 ± 0.12 A
Hardness (g)	228.24 ± 25.92 B	451.31 ± 11.49 A	441.42 ± 10.80 A	459.16 ± 18.17 A	450.56 ± 15.89 A
Chewiness (g)	179.83 ± 19.34 C	347.93 ± 9.46 B	398.93 ± 9.35 A	401.35 ± 5.11 A	383.68 ± 13.75 A
Cohesiveness (ratio)	0.88 ± 0.01 A	0.85 ± 0.02 A	0.87 ± 0.02 A	0.87 ± 0.01 A	0.88 ± 0.02 A
Springiness (mm)	0.95 ± 0.01 A	0.89 ± 0.01 B	0.96 ± 0.02 A	0.95 ± 0.02 A	0.96 ± 0.02 A
Cell density (cells/cm^2^)	53.00 ± 1.03 B	51.35 ± 2.05 B	56.85 ± 0.66 A	55.17 ± 1.86 AB	58.60 ± 1.20 A
Cell size (mm)	0.49 ± 0.01 A	0.39 ± 0.02 C	0.46 ± 0.01 B	0.51± 0.01 A	0.47 ± 0.02 AB
Mean cell area (%)	21.88 ± 1.33 A	20.12 ± 0.78 B	22.36 ± 0.12 A	23.15 ± 0.51 A	21.68 ± 0.21 B
IDF (%)	3.48 ± 0.11 B	4.76 ± 0.15 A	4.66 ± 0.21 A	4.72± 0.15 A	4.69 ± 0.26 A
SDF (%)	0.53 ± 0.01 B	2.50 ± 0.05 A	2.39 ± 0.15 A	2.43 ± 0.05 A	2.37 ± 0.06 A
TDF (%)	4.01 ± 0.10 C	7.26 ± 0.12 A	7.05 ± 0.25 A	7.15 ± 0.13 A	7.06 ± 0.25 A
Total starch (%)	43.82 ± 1.30 A	42.07 ± 0.21 B	41.86 ± 0.08 B	42.02 ± 0.06 B	41.76 ± 0.18 B
AUC	431.31 ± 21.4 A	337.27 ± 2.81 B	326.25 ± 12.26 B	332.58 ± 12.20 B	350.17 ± 11.79 B

Means ± standard deviation (*n* = 3). Values in the same row with different letters differ significantly (*p* < 0.05).

**Table 2:** Effect of enzyme combinations on the physical properties of CSBs.

Blocks	A	B	C	Specific Volume (mL/g)	Loaf Height (mm)	Moisture (%)	Hardness (g)	Springiness (mm)	Cohesiveness (ratio)	Chewiness (g)	Cells (Cells/cm^2^)	Cell Size (mm)	Cell Area (%)
Wheat flour	0	0	0	2.50	62.14	40.10	228.24	0.94	0.88	179.83	53.00	0.488	21.88
Buckwheat	0	0	0	2.19	57.65	41.15	401.31	0.89	0.85	307.97	51.35	0.39	20.12
1	6	70	35	2.25	57.18	45.18	398.42	0.92	0.87	334.43	49.18	0.55	23.82
2	6	70	60	2.50	60.88	47.69	271.46	0.95	0.88	230.43	46.68	0.70	26.98
3	6	120	60	2.31	61.28	46.31	391.76	0.97	0.89	349.32	50.10	0.65	23.90
4	6	120	35	2.30	59.68	44.25	364.13	0.98	0.88	314.25	51.50	0.55	24.13
5	10	70	35	2.28	54.79	46.31	390.35	0.97	0.97	343.27	49.63	0.53	23.18
6	10	120	35	2.27	57.51	43.63	356.43	0.96	0.90	327.09	52.50	0.49	23.85
7	10	120	60	2.22	57.88	44.33	379.78	0.95	0.90	359.09	51.50	0.51	22.05
8	10	70	60	2.29	54.91	46.28	346.86	0.98	0.91	305.13	49.67	0.60	22.29

All values are means. A (factor)—α-amylase; B (factor)—xylanase; C (factor)—cellulase; wheat flour—wheat flour CSBs; buckwheat—CSBs with 15 % buckwheat flour.

**Table 3:** Estimated regression coefficients of the factors of the physical properties of CSBs.

Coefficient Estimate	Specific Volume (mL/g)	Loaf Height (mm)	Moisture (%)	Hardness (g)	Springiness (mm)	Cohesiveness (Ratio)	Chewiness (g)	Cell Density (cells/cm^2^)	Cell Size (mm)	Cell Area (%)
Constant (β_0_)	2.26	56.88	45.65	331.68	0.96	0.90	308.18	49.52	0.58	22.88
Amylase (β_1_)	−0.02	−3.01	0.33	18.20	−0.01	NS	23.53	1.86	−0.11	−0.68
Xylanase (β_2_)	−0.05	−0.03	0.47	11.33	−0.02	NS	8.52	1.26	−0.06	−0.56
Cellulase (β_3_)	−0.07	−1.46	0.56	NS	−0.01	NS	11.31	−1.81	NS	−0.33
Amylase*Xylanase(β_12_)	0.02	0.12	−1.96	−2.14	0.02	NS	−3.21	3.66	−0.03	0.88
Amylase*Cellulase(β_13_)	0.03	0.11	0.66	−6.69	NS	NS	−12.11	NS	0.03	0.27
Xylanase*Cellulase(β_23_)	0.01	NS	0.26	−11.74	NS	NS	−3.08	−1.66	0.07	0.33
Amylase*Xylanase*Cellulase	0.02	NS	−0.44	−13.21	0.01	NS	−18.22	NS	−0.03	NS
R^2^	98.45%	91.85%	99.81%	98.18%	89.98%	40.82%	98.69%	91.19%	96.89%	89.85%

NS—no significant effect at level (*p* < 0.05); R^2^—adjusted square coefficient (describes the percentage of variability for which the model accounts); β_0_—global means of parameters; β_1_, β_2,_ and β_3_—regression coefficients corresponding to main factors; β_12_, β_13_, β_23_, —regression coefficients corresponding to interactions; ‘−’—negative effect.

**Table 4:** Effect of enzyme combinations on the chemical properties of CSBs.

Blocks	A	B	C	IDF %	SDF %	TDF %	Total Starch %	AUC
Wheat flour	0	0	0	3.48	0.52	4.01	43.82	491.30
Buckwheat flour	0	0	0	4.76	2.50	7.26	42.07	337.27
1	6	70	35	4.46	2.39	7.05	41.26	351.65
2	6	70	60	4.09	2.33	6.42	40.89	341.74
3	6	120	60	4.12	2.38	6.50	40.02	346.24
4	6	120	35	4.11	2.35	6.46	40.59	344.67
5	10	70	35	4.23	2.30	6.53	40.26	351.06
6	10	120	35	4.09	2.45	6.54	42.15	358.83
7	10	120	60	4.11	2.38	6.49	41.36	338.22
8	10	70	60	4.23	2.40	6.63	40.78	312.20

All values are means. A (factor)—α-amylase; B (factor)—xylanase; C (factor)—cellulase; wheat flour—wheat flour CSBs; buckwheat—CSBs with 15% buckwheat flour.

**Table 5:** Estimated regression coefficients of factors of the chemical properties of CSBs.

Coefficient Estimate	IDF %	SDF %	TDF %	Total Starch %	AUC
Constant (β_0_)	4.32	2.02	6.56	40.13	377.93
Amylase (β_1_)	−0.02	−0.01	−0.08	−0.01	−7.18
Xylanase (β_2_)	−0.13	0.03	−0.21	−0.02	−8.08
Cellulase (β_3_)	0.07	−0.11	−0.10	−0.08	−9.22
Amylase*Xylanase (β_12_)	NS	−0.08	−0.12	0.15	NS
Amylase*Cellulase (β_13_)	0.02	−0.02	−0.08	−0.26	8.19
Xylanase*Cellulase (β_23_)	0.01	NS	NS	−0.52	8.44
Amylase*Xylanase*Cellulase	0.05	−0.03	NS	NS	1.72
R^2^	91.11%	90.12%	96.58%	96.96%	95.23%

NS—no significant effect at level (*p* < 0.05); R^2^—adjusted square coefficient (describes the percentage of variability for which the model accounts); β_0_—global means of parameters; β_1_, β_2_, and β_3_—regression coefficients corresponding to main factors; β_12_, β_13_, β_23_,—regression coefficients corresponding to interactions; ‘ ‘−’—negative effect.

## Data Availability

The original contributions presented in the study are included in the article/Supplementary Material, further inquiries can be directed to the corresponding authors.

## Supplementary Materials

The following supporting information can be downloaded at: https://www.mdpi.com/article/10.3390/foods14152735/s1, Table S1. Function of enzymes (from Novozymes Biotechnology Company); Table S2. Description of experimental factors at two level.
