# Supplementary material for: Enzymes (α-Amylase, Xylanase, and Cellulase) in Steamed Buckwheat Buns: The Effects on Quality and Predicted Glycemic Response
Source: Foods. 2025 Aug 5;14(15):2735. doi: 10.3390/foods14152735 (PMC12346569; doi:10.3390/foods14152735)
Supplement: Supplementary file 1 [file foods-14-02735-s001.zip › foods-3739474-supplementary.pdf]

Supplementary Table S1. Function of enzymes (from Novozymes Biotechnology Company).

Enzymes	Component name	Activity	Fuction
Cellulast BG	endo-Glucanase	3500 EGU/g	hydrolyze (1,4)-β-D-glucosidic linkages in cellulose and other β-D-glucans
Fungamyl 2500 SG	α-Amylase	2500 FAU-F/g	hydrolyze (1,4)-α-D-glucosidic linkages in starch polysaccharides
Pentopan Mono BG	Xylanase (endo-1,4-)	2500 FXU-W/g	hydrolyze (1,4)-β-D-xylosidic linkages in xylans

EGU-Endo-Glucanase Units; FAU-Fungal Amylase Units; FXU-Fungal Xylanase Units

Supplementary Table S2. Description of experimental factors at two level.

Factor	(A) α – Amylase	(B) Xylanase	(C) Cellulase
Regular	-	-	-
Control	0	0	0
F1	-1	-1	-1
F2	1	-1	-1
F3	-1	1	-1
F4	1	1	-1
F5	-1	-1	1
F6	1	-1	1
F7	-1	1	1
F8	1	1	1

Amylase (-1, 1) – (6 ppm, 10 ppm); Xylanase (-1, 1) – (70 ppm, 120 ppm);
Cellulase (-1, 1) – (35 ppm, 60 ppm).
